# Favorable response to third-generation TKI furmonertinib in a patient with early-stage non-small cell lung cancer​ harboring rare compound EGFR mutations: Exon 18 G719C and Exon 20 S768I — A Case Report

**DOI:** 10.3389/fonc.2025.1714432

**Published:** 2026-01-16

**Authors:** Shihu Liu, Jinzi Zhang, Yanfeng Dong, Yongjie Wang

**Affiliations:** Qingdao University, Department Thorac Surg, Affiliated Hospital, Qingdao, China

**Keywords:** EGFR, EGFR Exon 18 G719C, EGFR exon 20 S768I, EGFR-TKI, furmonertinib, non-small cell lung cancer

## Abstract

EGFR Exon 19 deletions and exon 21 point mutations of EGFR are the most prevalent alterations in lung adenocarcinoma, and patients with these mutations derive substantial clinical benefit from EGFR tyrosine kinase inhibitors (TKIs). Nevertheless, the therapeutic efficacy of TKIs in rare compound EGFR mutations remains unclear. Here, we describe a case of early-stage non-small cell lung cancer (NSCLC) harboring a G719C+S768I compound mutation that achieved complete remission following treatment with furmonertinib. These findings suggest that furmonertinib may represent a promising therapeutic option to improve cure rates in this subset of patients.

## 1 Introduction

The prevalence of epidermal growth factor receptor (EGFR) mutations in non-small cell lung cancer (NSCLC) is notably high, ranging from 30% to 60% in Asian populations (1). In 2021, researchers at MD Anderson Cancer Center published a landmark study in *Nature*, in which they proposed a novel classification of EGFR kinase domain mutations into four distinct subtypes: classical-like, T790M-like, PACC, and exon 20 insertions (2). Among these, PACC mutations constitute a distinct subset, accounting for approximately 12.5% of all EGFR mutations, and include point mutations such as G719X, S768I, and L858R.

At the 2024 World Conference on Lung Cancer (WCLC), the FURTHER study evaluated the efficacy and safety of furmonertinib monotherapy as first-line treatment in patients with EGFR PACC–mutant NSCLC. Patients were randomized into two groups to receive furmonertinib 160 mg QD or 240 mg QD. The results showed that the objective response rate (ORR) was 81.8% in the 240 mg group and 47.8% in the 160 mg group (3).

## 2 Case presentation

65-year-old Chinese man was admitted on April 16, 2025, with a four-month history of cough with sputum and chest discomfort, which had worsened over the previous two weeks. He had a 30-year smoking history but quit 10 years earlier, and a 30-year history of alcohol use, also discontinued 10 years earlier. His medical history included cerebral infarction 10 years prior, leaving mild mobility limitations, and a 10-year history of hypertension managed with sustained-release nifedipine. He reported no family history of hereditary disease or infection. Over the past four months, he experienced unexplained chest tightness accompanied by cough and white sputum.

**Figure 1:**
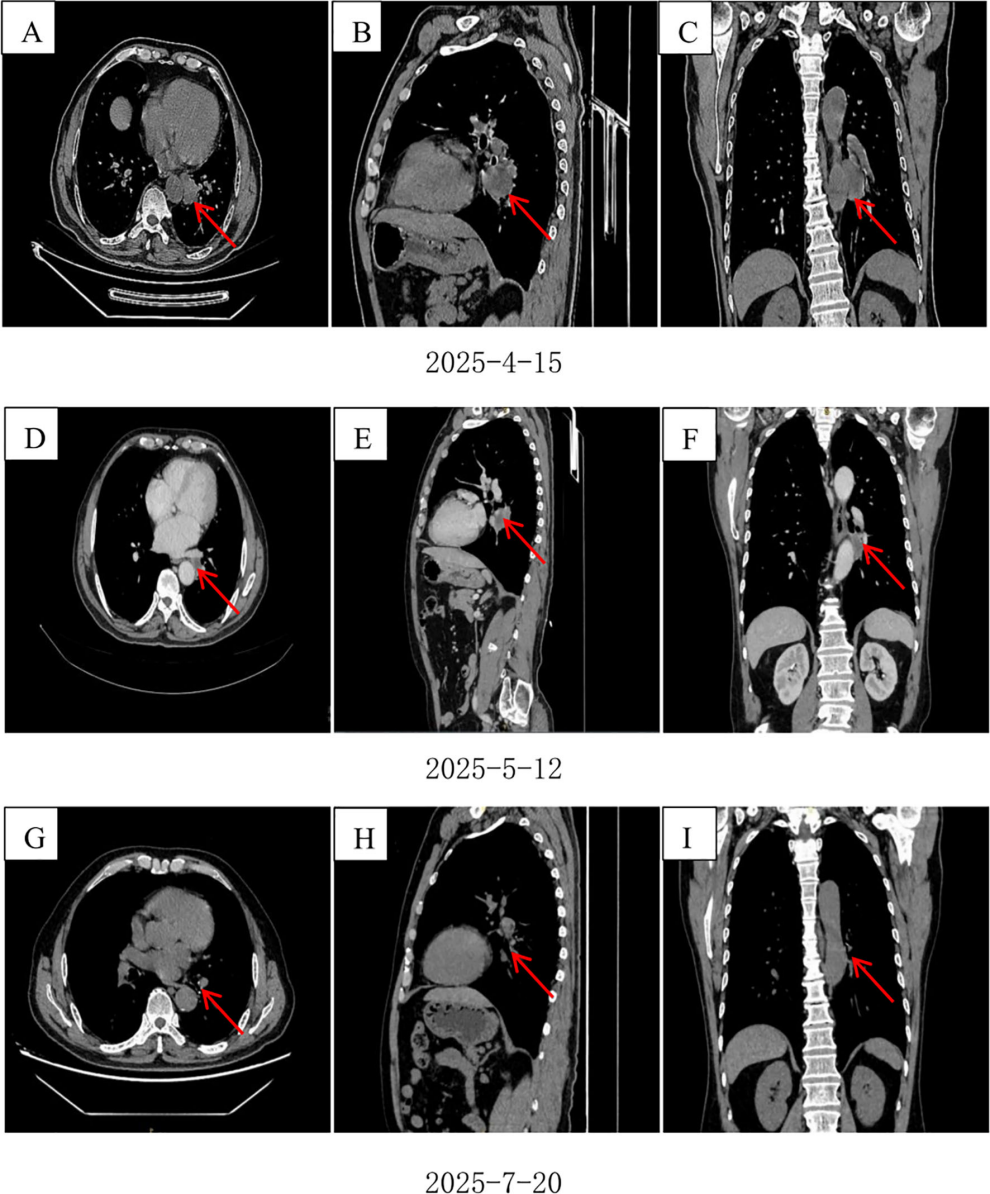
**(A)** Cross-section before treatment. **(B)** Sagittal plane before treatment. **(C)** Coronal surface before treatment. **(D)** Mid-treatment cross-section. **(E)** Mid-treatment sagittal plane. **(F)** Mid-stage coronal surface treatment. **(G)** Preoperative cross-section. **(H)** Preoperative sagittal plane. **(I)** Preoperative coronal plane.

**Figure 2:**
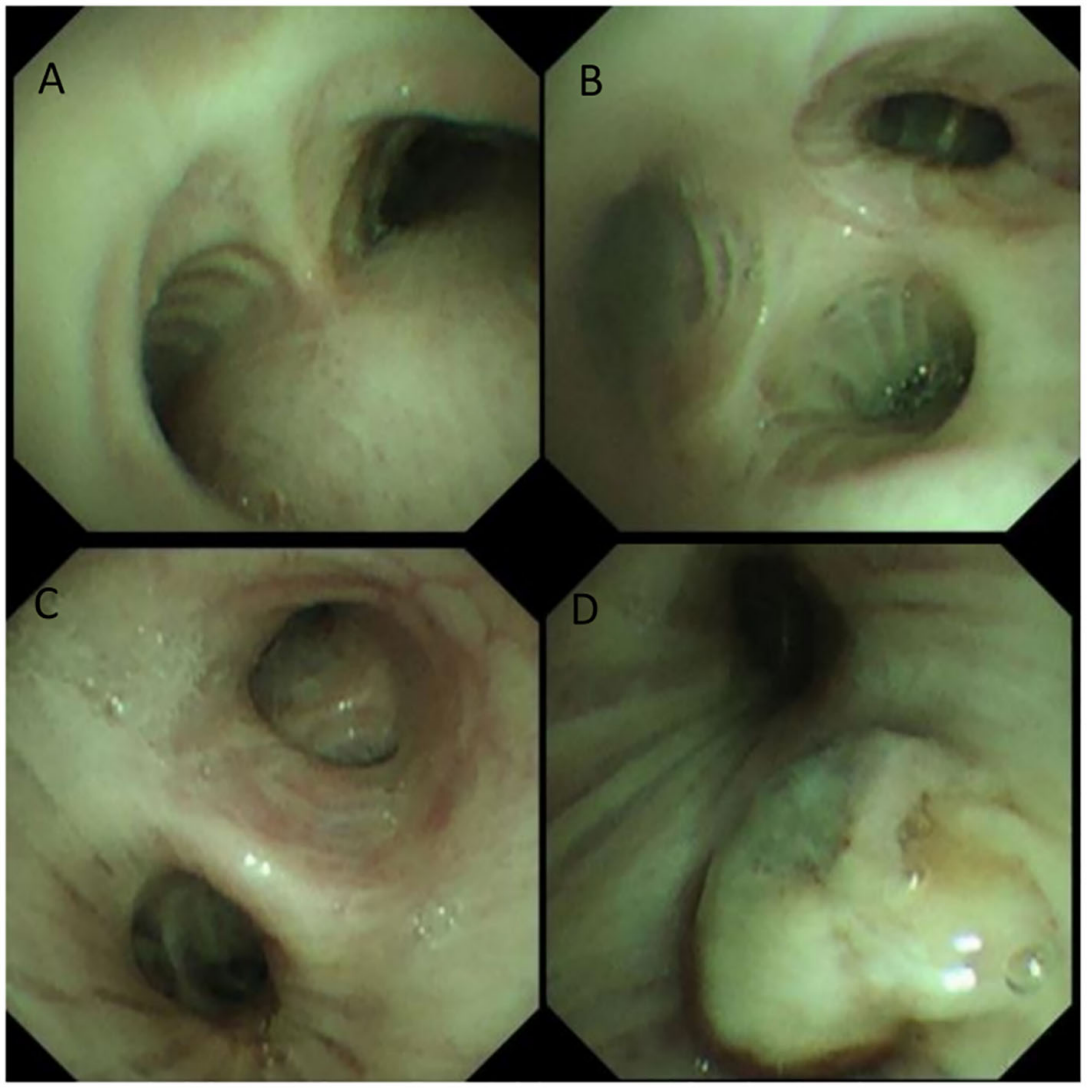
**(A)** tracheal carina **(B)** orifice of the right upper lobe bronchus **(C)** left main bronchus **(D)** orifice of the left lower lobe bronchus.

The patient presented to the Department of Thoracic Surgery at our hospital. A chest CT performed on April 15, 2025, revealed a 3-cm mass in the left lower lobe (**Figure 1**). Bronchoscopy on April 16, 2025, showed obstruction of the bronchial orifice of the dorsal segment of the left lower lobe by a neoplasm, while the remaining bronchi were patent and unremarkable (**Figure 2**). A biopsy specimen was obtained and submitted for pathological examination.

**Figure 3:**
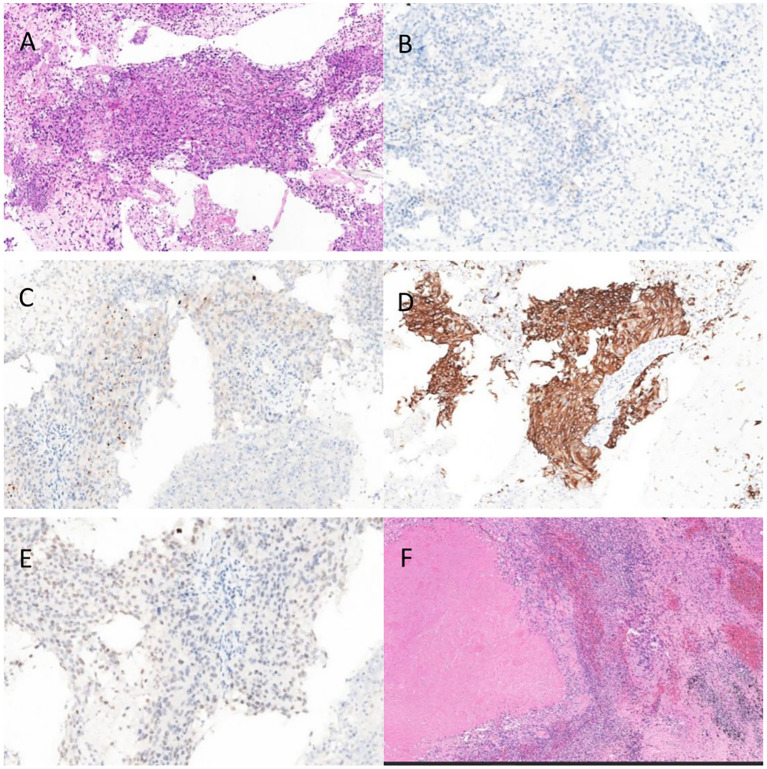
**(A)** Pre-EGFR TKI,HE **(B)** Pre-EGFR TKI P40 **(C)** Pre-EGFR TKI NaspinA **(D)** Pre-EGFR TKI CK5/6 **(E)** Pre-EGFR TKI TTF-1 **(F)** Post-EGFR TKI HE.

Pathology revealed non-small cell carcinoma with spindle and giant cell features. H&E–stained sections showed nested tumor growth with a high proportion of giant cells, abundant eosinophilic cytoplasm, variable nuclear size, irregular polygonal nuclear membranes, and increased nuclear-to-cytoplasmic ratio. Immunohistochemistry was positive for TTF-1, CK, CK5/6, GATA3, and Napsin A, and negative for P40, CgA, CD56, and Pax-8 (**Figure 3**).

**Figure 4:**
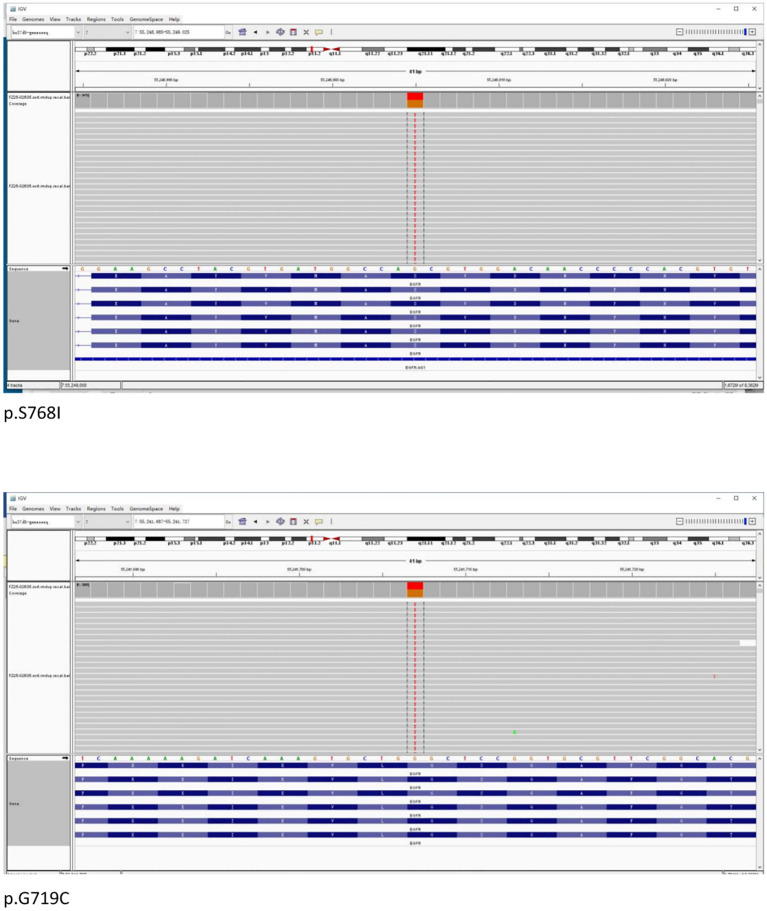
Next-generation sequencing revealed co-mutations in EGFR Exon 18 G719C and Exon 20 S768I.

Formalin-fixed paraffin-embedded (FFPE) samples with an 80% tumor cell percentage were prepared from the patient’s left lung biopsy tissue under bronchoscopy for next-generation sequencing (NGS) based on targeted DNA. NGS identified an EGFR exon 18 G719C mutation with an allele frequency of 44.24% and an EGFR exon 20 S768I mutation with an allele frequency of 47.71%. No additional mutations were found in other key driver genes (**Figure 4**).

Cranial MRA revealed a suspected aneurysmal protrusion in the C5 segment of the right internal carotid artery. Electrocardiography showed atrial flutter with tachycardia. After multidisciplinary discussion, the patient was deemed unfit for surgery. Oral Furmonertinib 160 mg QD, and cardiology managed his cardiac condition.

The patient started oral furmonertinib mesylate (160 mg QD) on May 1, 2025. Follow-up on May 12 showed marked tumor shrinkage without adverse effects, and therapy was continued (**Figure 1**). By July 1, imaging demonstrated complete remission. After multidisciplinary discussion, the patient underwent thoracoscopic left lower lobectomy with systematic lymph node dissection. Pathology revealed no residual viable tumor cells, with 40% necrosis and 60% stroma (fibrogranulation, histiocytic infiltration, and calcification) (**Figure 3F**). All lymph nodes were negative for metastasis, confirming CPR.

## 3 Discussion

The EGFR S768I mutation is a point mutation in exon 20, accounting for approximately 1.5%–3% of all EGFR mutations. At codon 768, the AGC sequence is replaced by ATC, resulting in a serine-to-isoleucine substitution (S768I) (4). The G719X mutation affects codon 719, replacing glycine with alanine (G719A), cysteine (G719C), or serine (G719S) (5). Different G719X variants show variable sensitivity to EGFR inhibitors, with G719C reportedly more responsive to erlotinib than G719A (6). Among rare compound mutations, G719X+S768I is the most frequently observed combination (7, 8).

A database analysis of 693 patients with rare EGFR mutations showed that those with major uncommon mutations (G719X, L861Q, and S768I) achieved a 77% objective response rate (ORR) and a median response duration of 16.6 months with afatinib treatment (9).

Third-generation EGFR TKIs, such as Osimertinib, have been used to treat NSCLC with rare EGFR mutations (10), though their efficacy is generally lower than that of Afatinib. Ongoing global studies, including the FURTHER trial, are evaluating Furmonertinib in EGFR PACC–mutant NSCLC, showing promising results (3). Furmonertinib, a novel third-generation EGFR TKI, is approved in China as first-line therapy for advanced EGFR-mutant NSCLC (11). Several multicenter trials are underway, including a phase III randomized, double-blind study (NCT04853342) and a study in EGFR-mutant, PD-L1–positive advanced NSCLC (NCT05255406). However, data on Furmonertinib in patients with rare or compound EGFR mutations remain limited.

Pan reported a case of NSCLC with brain metastases in a patient harboring the EGFR G719X/S768I compound mutation. After 14 months of treatment with furmonertinib 80 mg daily, bilateral pulmonary lesions showed marked regression, and cranial metastatic lesions were reduced (12).

Among 31 NSCLC patients with rare EGFR mutations and CNS metastases, Furmonertinib yielded an overall response rate (ORR) of 38.7%, with 50.0% ORR in those with compound rare mutations (13). Patients with Rare exon 18 G719A and exon 21 L833V compound EGFR mutations achieved progression-free survival for 12.5 months after first-line treatment with Furmonertinib (14).

Preclinical studies indicate that NSCLC patients with rare compound EGFR mutations show variable sensitivity to different TKIs (15). Chiu et al. reported that patients with the G719X+S768I compound mutation had longer progression-free survival (PFS) on EGFR-TKI therapy than those with single mutations (16). G719X frequently occurs with other rare mutations, and these compound mutations generally yield better outcomes than G719X alone (17, 18).

We report a case of early-stage NSCLC with a G719C+S768I compound mutation that achieved complete remission with Furmonertinib. This case may offer valuable insights into managing NSCLC patients with this rare compound mutation.

## Data Availability

The datasets presented in this study can be found in online repositories. The names of the repository/repositories and accession number(s) can be found below: Contact the corresponding author for information.
